# Cases of Pneumonia
^2^Read at the meeting of the Bath and Bristol Branch of the British Medical Association, held at Bristol, October 27th, 1909.


**Published:** 1909-12

**Authors:** Arthur Fells


### CASES OF PNEUMONIA.[2]

BY

ARTHUR FELLS, M.B., C.M. Edin.

THE following cases of pneumonia appear to be worth recording, the first because of the number of lesions, the other two on account of an infrequent sequel.

**Case 1.**—A well-nourished boy of 15. Was first seen on May 16th, 1909, with well-marked signs of early pneumonia of the right base. For four days he was delirious, with a temperature varying from 102° to 104°. On the seventh day the temperature fell to 100.7°, and there was every appearance of a crisis. At night, however, the temperature was 102.4°; next morning it was 103°, and a pneumonic patch at the left apex was found to have developed. On the thirteenth day the temperature fell to 98.6° and there was again every appearance of a crisis; but next

[2] Read at the meeting of the Bath and Bristol Branch of the British Medical Association, held at Bristol, October 27th, 1909.

day the temperature had risen to 102°, and a patch of pneumonia was found at the left base. From the sixteenth to the eighteenth days the disease appeared to be resolving by lysis, and there was a general improvement in every respect. On the nineteenth day the temperature assumed a zigzag character, varying from 98.7° in the morning to 101.6° at night, each day mounting slightly higher. The old area of dullness at the right base was found to be rising higher, but the breath sounds were readily audible right down to the base of the lung. The apex beat was not displaced, but a loud systolic mitral murmur had developed. The boy's general condition was very bad, he was wasting rapidly, and although not delirious, was very childish. Pus having been detected in the right thorax on the twenty-second day, a rib was resected, and a large quantity of pus evacuated. For a few days the patient seemed to improve, but the pulse rate kept at 130 to 150, the respirations at 30 to 40, and the temperature remained above normal. For some days the boy had complained of pain in the left hip, stating that he had sprained it shortly before the onset of the pneumonia. By about the twenty-eighth day the hip, which had assumed a position of semi-flexion and abduction, was found to be tense and swollen, especially on the adductor aspect. It rapidly became extremely tender, but no definite fluctuation could be detected. The discharge from the empyema had almost ceased, but the urine, which had been examined several times with negative results, was now found to contain granular casts, blood and much albumin. On the thirty-third day the patient was removed to the hospital, where Mr. Lansdown kindly saw him late in the evening, and at once performed an arthrotomy of the left hip, evacuating an excess of blood-stained synovial fluid from the joint but no pus. On the removal of the clips from the hip incision there was an escape of pus from the wound, which was found to yield a pure culture of the pneumococcus. The discharge from the hip ceased in a few days, but still the boy did not improve satisfactorily until the forty-third day, when there was a gush of pus from the thoracic incision (the tube had been removed some days previously), whereupon the temperature fell to normal and remained so. Through most of the illness the boy had taken nourishment well, but he had lost two stones in weight, and the rapidity of the wasting was shown by the development of very prominent striæ atrophicæ across the loins, on the right buttock and thigh, and on the right arm. When at the Convalescent Home he lost all trace of the nephritis, the left hip appeared to be as sound as the right, and he rapidly put on flesh ; but the pulse rate was still 100, and the mitral murmur was still present. During two months in the country the boy more than regained his lost weight, grew nearly three-quarters of an inch in height, and on his return was found to have a pulse rate of 75 and normal heart sounds.

The pneumococcus was thus responsible for three distinct and consecutive pneumonic attacks—an empyema, a nephritis and an arthritis. The nature of the cardiac condition was not clear. It is very improbable that it could have been an endocarditis, and the murmur bore no resemblance to a pericardial rub. Its history suggested a murmur due to dilatation, but if so, other physical signs of that condition were lacking.

As regards nephritis in pneumonia, Dr. Hector Mackenzie,[1] during the discussion upon that disease before the Royal Society of Medicine in 1907, quoted twelve instances of the former in 750 cases of the latter, and of the twelve, eight proved fatal. The same speaker referred to sixty-three cases of pneumococcal arthritis occurring in pneumonia collected from several sources, and of these forty-one proved fatal.

For statistical purposes this form of arthritis occurring in pneumonia should be distinguished from the corresponding joint condition occurring apart from any general infection.

In the following cases pneumonia was followed by thrombosis in the veins of the left leg.

**Case 2.**—A fragile man, of about 40, was taken ill with what was probably influenza on February 21st, 1908. When first seen, on the 27th, pneumonia of the right base was developing. He was very ill for five days, resolution occurred by lysis, the temperature falling from 103·5° to normal in five days. On March 17th, twenty-five days after having first taken to bed, he got up for the first time, and had just time to put on his trousers when he was seized with violent pain in the left leg, which almost caused him to faint. He at once returned to bed, and when seen a few hours later, the left leg and thigh were much swollen, very hard and tender. The superficial veins stood out in dark blue projections, acutely dilated. The circumference of the left calf was $2\frac{1}{4}$ inches, and the left thigh $1\frac{3}{4}$ inches in excess of the other limb. Nine days later the affected limb was still one inch larger than its fellow, the internal saphena vein could be felt as a thick cord, and there was much tenderness over the deeper veins. For several weeks there was much weakness and some swelling of the limb.

**Case 3** presents several points similar to the foregoing. On March 2nd, 1908, a robust woman, of about 60, fell down a flight of stairs and badly bruised the lower ribs of the right side. Five days later pneumonia developed in the right base.

[1] *Proc. Roy. Soc. Med.*, 1908, i, Med. Sect., p. 14.

The symptoms and physical signs were typical of lobar pneumonia, but again resolution was by lysis. Twenty-five days after the original accident the patient got out of bed for the first time, and was suddenly seized with violent pains in the left leg, accompanied by swelling, the left calf being 1¼ inch more in circumference than the right. The swelling was firm, resistant and very hard, and the pain remained severe for several days, then gradually diminished, but the limb still needs the support of a bandage.

Many books make no reference to thrombosis as a complication or sequel of pneumonia, and personally I have not met with it in some 300 cases treated in Indian hospitals. Dr. Mackenzie, on the occasion already referred to, spoke of it as occurring once in every 150 cases, and usually in the left leg. In the two instances given above it will be noticed that the pneumonia followed some other condition, that it involved the right base and resolved by lysis. The thrombosis appeared with startling suddenness when the patient first stood up after three weeks in bed, and when convalescence from the pneumonia was well established. The swelling was peculiarly hard, and there was no pitting on pressure.

